# Effects of restoration duration on plant community and soil chemical stoichiometry characteristics in mine waste dumps

**DOI:** 10.3389/fpls.2025.1693953

**Published:** 2025-12-10

**Authors:** Yazhou Shao, Hejun Zuo, Yunxia Ma, Xiaolu Ma, Gangtie Li

**Affiliations:** 1 College of Desert Control Science and Engineering, Inner Mongolia Agricultural University, Hohhot, China; 2 State Key Laboratory of Water Engineering Ecology and Environment in Arid Area, Inner Mongolia Agricultural University, Hohhot, China

**Keywords:** open-pit coal mine, waste dump, restoration period, vegetation characteristics, soilstoichiometric ratios

## Abstract

Taking the dump slopes and platforms with different restoration years (2Y, 6Y, 10Y, 12Y) in Manlailiang Open-pit Coal Mine as the research objects, combined with the unmined original landform (CK), the effects of restoration years on plant communities and soil stoichiometric characteristics were explored. The vegetation characteristics were investigated by ‘ large quadrat nested small quadrat ‘, and the soil nutrients and stoichiometric ratio were measured in 0–30 cm soil layer, combined with statistical methods such as two-factor analysis of variance. With the increase of restoration years, the species increased, the importance value of *Medicago sativa* decreased, and the native perennial plants became the dominant species (shady slope was better than sunny slope). Organic carbon increased, nitrogen showed ‘ V ‘ shape (12Y super CK), and phosphorus was stable; the topographic measurement of 12Y is close to CK, and the vegetation and soil are synergistically close to CK. The restoration is divided into three stages (initial 2Y-6Y, middle 10Y, late 12Y), 12Y near-natural state (as a key node), and the shady slope recovery is the best, which can provide a basis for differentiated restoration.

## 1 Introduction

In the past 10 years, the strategic focus of China ‘s coal industry has accelerated to the west. Shanxi, Shaanxi, Inner Mongolia, Xinjiang and other central and western coal reserves have become the concentrated distribution area of large coal bases (Wang et al., 2024a; Zhu et al., 2024). Among them, the coal reserves of Inner Mongolia Autonomous Region ranks second in the country. The coal resources in the country are characterized by shallow burial and large reserves. The number of open-pit coal mines ranks first in the country and is an important energy security base in China. Ordos City is the core distribution area of open-pit coal mines in the autonomous region (Yang et al., 2023). Taking Manlailiang Open-pit Coal Mine (located in Nalintaohai Town, Yijinhuoluo Banner, Ordos City, with an annual output of 2.4 million tons of coal) as an example, the dump formed in the mining process has problems such as steep slope, exposed surface and loose soil structure (Xu et al., 2025), which makes it difficult for vegetation to settle naturally. At the same time, the heavy metals in the slag may pollute the surrounding water environment and inhibit the growth of plant roots. The fine particles are easy to cause atmospheric dust. The slope also faces the risk of geological disasters such as landslide collapse and soil erosion, and the ecological restoration is extremely difficult.

Open-pit mining is one of the most severe activities of human beings to destroy the surface ecosystem. Large-scale excavation and dumping operations not only directly damage the soil vegetation system (Fan et al., 2021), but also change the key ecological processes such as regional hydrological cycle and material cycle, thus threatening the stability of the ecosystem and the protection of biodiversity (Tang et al., 2021) Among the many ecological problems caused by open-pit mining, soil nutrient depletion and severe vegetation degradation caused by soil collapse and soil erosion are particularly prominent. As the core carrier of plant growth and development, soil nutrient status directly determines plant survival, growth and community construction (Cortois et al., 2016); plants can feed back to the soil through litter return, root exudates input and rhizosphere microbial regulation, and participate in soil organic matter accumulation, nutrient cycling and structural improvement. They form a close relationship of ‘ soil nutrient supply-plant growth feedback-soil quality improvement ‘, and jointly support the net primary productivity and ecological service function of the ecosystem (Liang et al., 2019; Wang and Mu, 2012; Yang and Zhao, 2020).

The three basic nutrient elements of carbon (C), nitrogen (N) and phosphorus (P), which are dependent on plant growth, have synergistic effects in physiological metabolism (Zhou et al., 2018; Gou et al., 2020).Their content and ratio in soil (C/N, C/P, N/P) are key indicators to measure the composition and quality of soil organic matter, and are also the core basis for revealing plant nutrient utilization efficiency and growth limiting factors (Cheng et al., 2010). Specifically, the soil C/N ratio reflects the mineralization rate of organic matter (Fan et al., 2019). When C/N > 25, the organic matter accumulation rate exceeds the decomposition rate (Bui and Henderson, 2013), and the soil carbon pool shows an increasing trend. When C/N is 12-16, organic matter is mainly decomposed, which can release available nutrients for plants. Soil C/P ratio reflects the ability of microbial mineralization of organic matter to release phosphorus (Yuan, 2024), which is directly related to the bioavailability of soil phosphorus. Soil N/P ratio, as a diagnostic index of nitrogen saturation, can be used to determine the critical point of plant growth limited by nitrogen or phosphorus (Huang and Yuan, 2020). The dynamic changes of these three stoichiometric ratios not only depict the soil nutrient balance, but also indirectly reflect the material exchange efficiency between vegetation and soil, which is an important quantitative basis for evaluating the process of ecosystem restoration.

As a typical derivative landform of open-pit coal mining (loose accumulation formed by stripping rock and soil handling and accumulation), the ecological restoration of the dump is a key link in the sustainable development of the mining area (Bi et al., 2021; Li et al., 2019). At present, China ‘s research on mining dumps mostly focuses on a single terrain (such as only focusing on platforms or slopes). However, due to serious soil erosion and poor soil nutrients, the remediation needs of dump slopes are significantly different from those of platforms, and it is difficult to directly apply the same remediation measures (Ji et al., 2021; Deng et al., 2021; Xue et al., 2021). Based on this, this study proposes the following assumptions: (1) With the increase of restoration years, the plant community (species diversity, dominant species) and soil stoichiometric ratio of the dump will be restored synergistically. At 12 years, it approaches the unexploited original landform (CK), and soil organic carbon and total nitrogen are the core driving factors; (2) Under the same year, the vertical consistency of plant diversity and soil stoichiometric ratio on the shady slope was better than that on the sunny slope. Due to manual management, the soil carbon and nitrogen-carbon and phosphorus accumulation on the platform was more synergistic, and the vegetation succession was faster.

In order to verify the above assumptions and scientifically formulate differentiated restoration strategies, this study takes the slopes and platforms of the dumps with different restoration years (2010, 2012, 2016 and 2020, corresponding to the restoration years of 12Y, 10Y, 6Y and 2Y) in Manlailiang Open-pit Coal Mine as the research object, and takes the unexploited original landform (CK) as the control. Through on-site investigation and sample determination, the evolution of plant communities (species composition, important value, diversity) and soil physical and chemical properties (organic carbon, total nitrogen, total phosphorus content and stoichiometric ratio) under different restoration years was analyzed. The purpose of revealing the correlation mechanism between vegetation characteristics and soil stoichiometric ratio is to provide data support for the precise ecological management and sustainable development of the dump in Manlailiang Open-pit Coal Mine.

## 2 Materials and methods

### 2.1 Overview of the study area

#### 2.1.1 Geographical location

**Figure 1:**
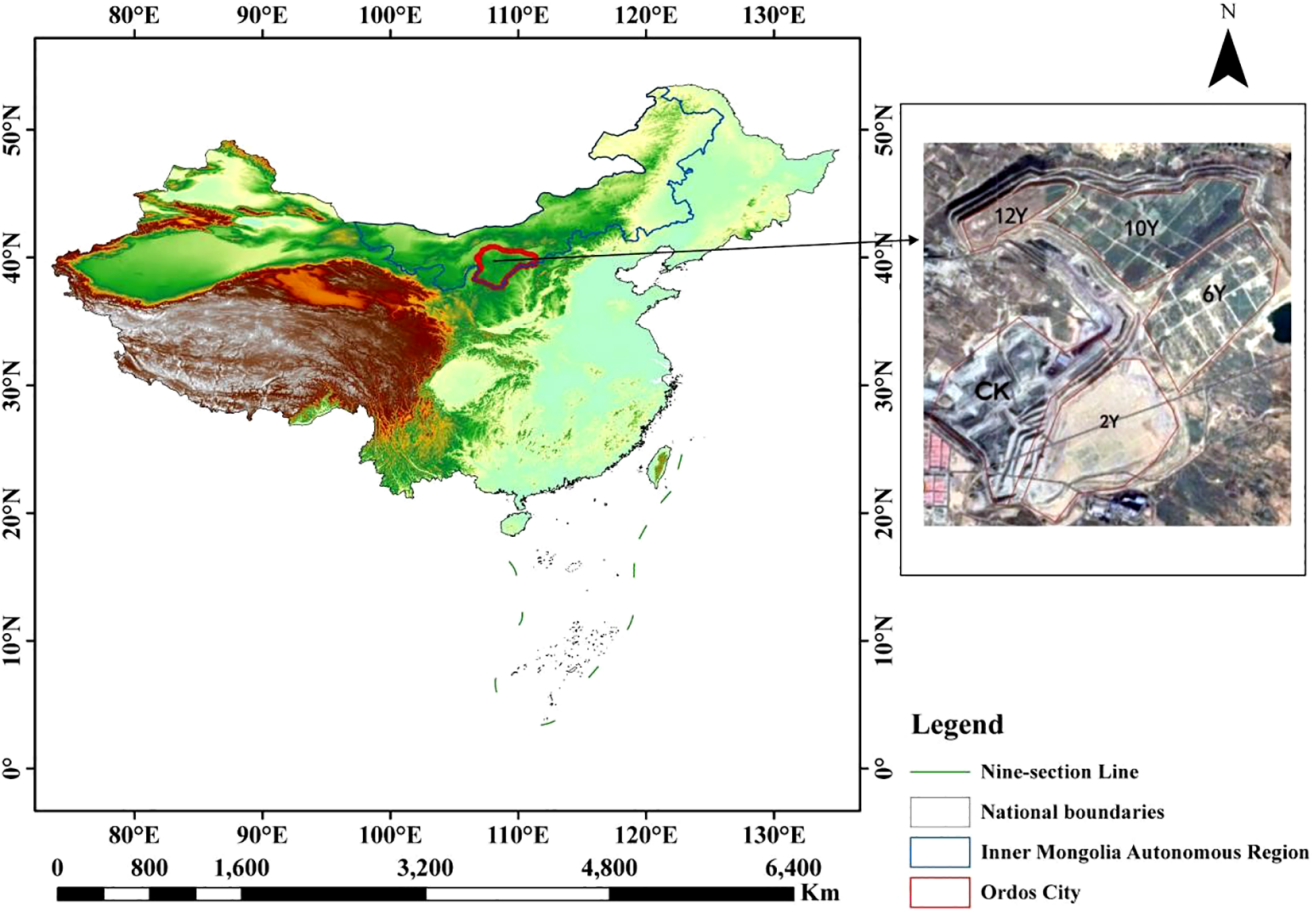
Overview map of the study area.

As shown in **Figure 1**, the test area is located in Manlailiang Open-pit Coal Mine, Nalintaohai Town, Yijinhuoluo Banner, Ordos City, Inner Mongolia. It is located in the transitional zone between the northeastern Ordos Plateau and the Loess Plateau. The geographical coordinates are 38° 56 ‘ -39° 49 ‘ north latitude and 108° 58 ‘ -110° 25 ‘ east longitude. The region is temperate continental climate, annual rainfall is 340-420mm (decreasing from southeast to northwest), annual average temperature is 6.2°C, frost-free period is 130–140 days, annual sunshine is 2700-3100h, annual evaporation is 2163mm (about 7 times of rainfall). The total amount of water resources in the whole banner is 331.1231 million m3, including 223.9094 million m3 of surface water and 128.8425 million m3 of groundwater. The soil is mainly sandy soil, zonal distribution of coarse bone soil, chestnut soil, meadow soil, saline soil and fluvo-aquic soil; the vegetation is a temperate semi-arid steppe, with perennial grasses as the core. Compositae, legumes and small shrubs are widely distributed, of which *Artemisia ordosica* accounts for about 60%, and the vegetation types in the Mu Us Sandy Land are abundant.

#### 2.1.2 Mining and reclamation of mining area

The annual output of coal in Manlailiang Open-pit Coal Mine is 2.4 million tons. Since 2010, the restoration work of dump has been started, and the restoration measures have been gradually optimized with the development of technology. In the early stage, the single planting mode of *Medicago sativa* was adopted. In the later stage, the sand barrier of *Salix psammophila* was added to the new dump slope, and *Pennisetum giganteum*, *Xanthoceras sorbifolium Bunge*, *Morus alba* and so on were supplemented in the platform area. However, the alfalfa planting mode is still continuously applied on the slopes and platforms of the dumps with different restoration years (the earliest restoration area has been restored for more than 20 years, with a total planting area of more than 3,000 mu), and the supporting sprinkler irrigation system is irrigated regularly, and the alfalfa is mowed every autumn. The coal mine adopts the operation mode of “ mining while reclamation, “ and the base of the dump is the waste such as rock, bedrock debris and coal gangue produced by mining. The platform is covered with the original landform topsoil (the thickness of the soil is about 2m), and the slope is maintained at 20° -30° (length of about 20m) after artificial slope, and the original landform topsoil (the thickness of the soil is about 0.6m) is also covered.

#### 2.1.3 Sample plot setting

The site selection of this study is based on the core principles of ‘ consistency of restoration measures, terrain representativeness, and data comparability ‘:

1. Consistency of restoration measures: The selected dumps with different restoration years (2Y, 6Y, 10Y, 12Y) all continued the restoration mode dominated by alfalfa, and the management measures such as sprinkler irrigation and mowing were unified to eliminate the interference of restoration technology differences on vegetation-soil evolution.
2. Terrain representativeness: referring to the typical terrain structure of the dump in the mining area, three core micro-terrain units of ‘ sunny slope-shady slope-platform ‘ are selected respectively, and the slope gradient (20° -30°) and the thickness of the platform covering soil (about 2m) all meet the mining area reclamation engineering standards, avoiding abnormal data caused by extreme terrain (such as steep slopes, low-lying water areas);
3. Spatial proximity: All plots are located in the same mining area (distance ≤ 5km) of Manlailiang Open-pit Coal Mine, and the background conditions such as climate and parent material are consistent, so as to ensure that the recovery period is the core variable driving vegetation-soil change.

The survey was carried out in September 2022. Four dumps with different restoration years (2Y: reclamation in 2020, 6Y: reclamation in 2016, 10Y: reclamation in 2012, 12Y: reclamation in 2010) were taken as the research objects, and the unexploited original landform was taken as the control (CK). In the sunny slope, shady slope, alfalfa planting platform and CK area of each recovery year dump, one representative sample plot was set up respectively. Three 5m × 5m arbor/shrub quadrats were set up in each sample plot with equal spacing (interval 10m) along the contour line direction. One 1m × 1m herb quadrat was set up in the four corners and the center of each 5m × 5m quadrat (15 1m × 1m herb quadrats in each sample plot).

#### 2.1.4 Discussion on the impact of spatial heterogeneity

Although this study reduced the influence of micro-scale heterogeneity through multi-point repetition in the plot, it is still necessary to objectively understand the potential effect of spatial variability on the results: 1) Slope position heterogeneity: due to more serious soil erosion, the soil nutrient (especially organic carbon and total nitrogen) content may be lower than the middle and lower slope positions, which may lead to the overall nutrient mean of the slope slightly lower than the actual level; however, this study has balanced the difference of slope position to a certain extent through the unified distribution of ‘uphill-middle slope-downhill ‘. 2) Heterogeneity of platform covering soil: Although the overall thickness of platform covering soil is about 2m, there may be a thickness fluctuation of ± 0.2m in some areas, which may lead to a slight difference in nutrient content of deep soil (20-30cm). However, the platform soil is mainly covered by artificial soil, and the uniformity of parent material is high, which is weak. 3) Heterogeneity of species distribution: The patchy distribution of *Medicago sativa* and native plants may lead to local fluctuations in community diversity index, but the interference of species patch distribution can be reduced by calculating the mean value of 15 1m × 1m herb quadrats.

In general, this study has controlled spatial heterogeneity to the greatest extent through standardized site selection, nested sampling and micro-topography records; if similar studies are carried out in the future, stratified random sampling (stratified by slope position and overburden thickness) can be further used to increase the number of quadrats (5-6 5m × 5m quadrats per restoration year), and geostatistical methods (such as semi-variance analysis) can be introduced to quantify spatial heterogeneity and further improve the robustness of the results.

### 2.2 Sample collection and determination

#### 2.2.1 Community characteristics

(1) The importance value is a comprehensive quantitative indicator used to represent the status and role of a species within a community.

(1) Importance value=(relative coverage+relative density+relative frequency)/3

Relative cover: the percentage of a species’ cover relative to the total cover of all species in the community.

Relative density: the percentage of individuals of a species relative to the total number of individuals of all species within the plot.

Relative frequency: the percentage of sampling plots in which a species is present relative to the total number of sampling plots.

(2) The community diversity indices in this study are calculated using four commonly employed diversity indices.

Margalef richness index

(2) $$\text{R}=\frac{\left(\text{S}-1\right)}{\ln\text{N}}$$

Shannon-Wiener diversity index

(3) $$\text{H}=-\sum_{\text{i}=1}^{\text{n}}{\text{p}}_{\text{i}}\ln{\text{p}}_{\text{i}}$$

Pielou’s evenness index

(4) E=H∕lnS

Simpson’sdominance index

(5) $$\text{D}=1-\sum_{\text{i}=1}^{\text{n}}{\text{p}}_{\text{i}}^{2}$$

In the equation, Pi represents the ratio of the number of individuals of species i in a community to the total number of individuals, S denotes the number of species in the population, and N represents the total number of individual plants in the population.

#### 2.2.2 Determination of soil physicochemical properties

Soil samples were collected from the same locations as the community plots. In each quadrat, the surface litter was gently scraped off, and approximately 1 kg of soil was collected from each of the following depths: 0–10 cm, 10–20 cm, and 20–30 cm. Large stones, tree roots, and other debris were removed, and the soil from each layer was thoroughly mixed. Repeated sampling was conducted, and the samples were then numbered, sealed in self-sealing bags, and transported to the laboratory for nutrient content analysis. All soil samples were collected within a short period, and no rainfall occurred during the sampling period. Soil organic matter (SOM) was determined using the potassium dichromate digestion method with external heating; total nitrogen (TN) was measured by potassium dichromate oxidation with external heating; and total phosphorus (TP) was quantified using the sodium hydroxide fusion method followed by the molybdenum-antimony anti-colorimetric method.

### 2.3 Data analysis

Excel 2021 was used for data collation and preliminary calculation, IBM SPSS Statistics 27.0 and Canoco 5.0 were used for statistical analysis, and Origin 2022 was used for chart drawing.

The specific statistical methods are as follows:

Difference significance test: Two-way ANOVA was used to analyze the independent and interactive effects of restoration years and topography (aspect/platform) on soil organic carbon, total nitrogen, total phosphorus content and their stoichiometric ratios (C/N, C/P, N/P). If the interaction is significant, a simple effect analysis is performed; if not significant, the Tukey HSD method was further used for *post-hoc* multiple comparisons to test the significance of differences between different recovery years or terrains (α = 0.05).

Comparison between groups: For the same recovery period, the terrain (sunny slope/shady slope/platform) and soil layer (0-10/10-20/20-30cm) ‘ were compared by independent samples T-test.

Correlation analysis: Pearson correlation coefficient was used to analyze the correlation between soil stoichiometric ratios at different soil depths (0–10 cm, 10–20 cm, 20–30 cm). The significance level was set as * p < 0.05, **p < 0.01, *** p < 0.001.

Principal component analysis (PCA): Canoco 5.0 was used to perform principal component analysis on vegetation diversity index (Margalef richness index, Shannon-Wiener diversity index, Pielou evenness index, Simpson dominance index) and soil stoichiometric ratio (C/N, C/P, N/P) to reveal the synergistic relationship between vegetation and soil nutrients.

Comprehensive evaluation: Based on the results of principal component analysis, combined with the variance interpretation rate, the ecological restoration effects of different restoration years and terrain combinations were comprehensively evaluated.

## 3 Results

### 3.1 Vegetation community characteristics of tailings dumps with different restoration durations

**Figure 2:**
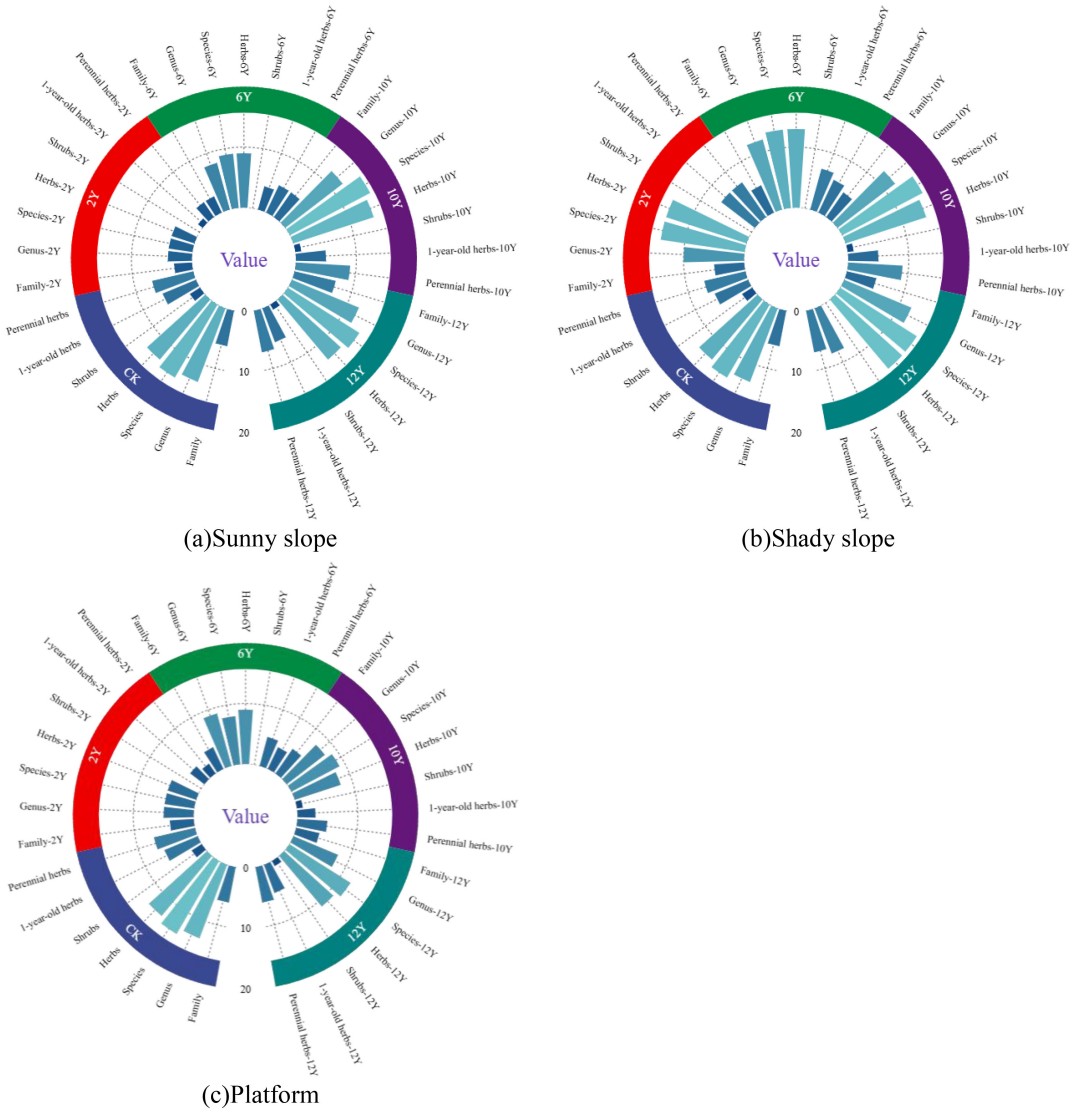
Changes in plant species at the spoil heap under different restoration durations. **(a)** The plant community characteristics of the sunny slope of the dump; **(b)** The plant community characteristics of the shady slope of the dump; **(c)** Plant community characteristics of dump platform.

Data from (**Figures 2a, b**) indicate that a total of 60 plant species, belonging to 17 families and 45 genera, were identified on the slope of the mine waste dump. Among these plants, the main groups are the *Asteraceae, Poaceae, Fabaceae*, and *Amaranthaceae*, with 22 species in *Asteraceae*, 10 in *Poaceae*, 8 in *Fabaceae*, and 3 in *Amaranthaceae*. In terms of plant types, herbaceous species dominate the slope of the coal mine waste dump, accounting for 56 species, over 90% of the total; in contrast, erect shrubs and subshrubs consist only of *Artemisia ordosica*, *Lespedeza potaninii*, and *Amorpha fruticosa*. With the increase in restoration years, species in the *Scrophulariaceae*, *Euphorbiaceae*, and *Tribulaceae* gradually disappear, while species from the *Rosaceae*, *Orobanchaceae*, and *Apocynaceae* are newly introduced. During vegetation succession, species in the *Asteraceae*, *Poaceae*, and *Fabaceae* consistently maintain dominance, with the *Asteraceae* having the highest proportion. Moreover, the composition in terms of families and genera also changes with the restoration period, with their distribution shifting from a higher number on the sunny slope in the early stages of restoration to a greater number on the shady slope over time. The number of species in each plot increases with restoration time, leading to a more diverse species composition and changes in dominant species. Artificial vegetation shows a distinct trend of degradation, with native plants gradually gaining dominance, and community structure progressing towards stability. It is noteworthy that within the same restoration period, the dominant species vary between different slope aspects; furthermore, with increased restoration time, both the growth status and survival number of plants on the shady slope are superior to those on the sunny slope.

Data from (**Figure 2c**) indicate that a total of 19 plant species, belonging to 5 families and 15 genera, were recorded on the coal mine dump platform. The four dominant families, consistent with those found on the slopes, were *Asteraceae* (8 species), *Poaceae* (6 species), *Amaranthaceae* (3 species), and *Fabaceae* (1 species). In terms of plant types, herbaceous plants constituted the primary species on the platform, with a total of 18 species accounting for over 90% of the flora; in contrast, erect shrubs and subshrubs were represented solely by *Artemisia ordosica*. With increasing restoration age, the number of *Asteraceae* and *Poaceae* species gradually increased and became dominant. During the process of vegetation succession, noticeable changes occurred in the composition of families, genera, and species. Each plot exhibited a continuous increase in species number over time, leading to a more diversified species composition, while the artificially established vegetation showed a trend of degradation.

### 3.2 Analysis of the importance value of slope vegetation in tailings dumps with different restoration durations

#### 3.2.1 Analysis of the importance value of slope vegetation in tailings dumps with different restoration durations

Analysis of the importance value distribution in **Table 1** reveals that *Medicago sativa* is present on the slopes of the tailings dump across four different restoration durations, although its importance value gradually decreases with increasing restoration age. In the initial stages of restoration, *Medicago sativa* exhibits the highest importance value, followed closely by *Imperata cylindrica* and *Salsola collina*. As the restoration process advances, native geomorphic species progressively invade and assume dominance. At 6 years of restoration, annual and biennial plants such as *Eragrostis pilosa* and *Grubovia dasyphylla* have relatively high importance values, with a few perennial species beginning to appear. By 12 years, the importance values of perennial species such as Aster altaicus, A*rtemisia ordosica, Leymus chinensis*, and *Elymus dahuricus* have significantly increased, leading to a shift in community structure where perennial native species gradually replace the artificially seeded *Medicago sativa* as the dominant life form on the slopes. **Table 1** also shows that the restoration outcomes on different slope aspects at the tailings dump vary even under the same restoration age. Specifically, at 12 years, the proportion of importance values attributed to perennial species reaches 54.56% on the shady slopes and 56.60% on the sunny slopes, representing increases of 4.77% and 18.66%, respectively, compared to the 6-year restoration period. Overall, perennial species better maintain population stability and are less influenced by environmental disturbances. The shifts in species life form composition clearly reflect the dynamics of species competition and succession during vegetation restoration, while the invasion of native geomorphic species further drives ecosystem adjustments, guiding the community toward a stable state.

**Table 1:** Vegetation importance values on spoil dump slopes under different restoration durations.

Number	Plant name	Important value%								
		2YS	2YN	6YS	6YN	10YS	10YN	12YS	12YN	CK
1	*Grubovia dasyphylla*			3.79	12.11	7.57			3.83	5.51
2	*Leymus chinensis*			2.6			7.79		17.73	
3	*Medicago sativa* L.	26.06	18.17	15.31	12.06	5.81	11.01	9.46	6.62	9.93
4	*Pennisetum flaccidum*	5.31	3	13.65	18.46		2.67		3.29	9.63
5	*Potentilla sericea*							2.22		
6	*Erigeron canadensis*								3.79	
7	*Aster altaicus*					6.75	10.28		12.58	3.32
8	*Elymus dahuricus*			7.01			5.73	5.02	9.46	
9	*Artemisia frigida*							2.13		
10	*Artemisia annua*		9.22		6.08	6.15		5.8	6.87	
11	*Imperata cylindrica*	39.88	9.13							
12	*Artemisia scoparia*		13.18		8.42				3.37	
13	*Silene songarica*							2.17		
14	*Incarvillea sinensis*							2.21	6.4	
15	*Oxytropis racemosa*							2.3		2.96
16	*Artemisia argyi*		4.01							
17	*Xanthium strumarium*		2.71						1.61	3.07
18	*Vicia amoena*							2.17		
19	*Artemisia sieversiana*		5.7				5.45		7.24	6.18
20	*Dracocephalum moldavica*							2.22		
21	*Leymus secalinus*			6.41		9.98	4.97	11.91		9.47
22	*Salsola collina*	28.75	12.2	20.47		16.1	8.06	6.83		19.37
23	*Melilotus suaveolens*						2.87		1.61	
24	*Bidens parviflora*						9.12		8.85	
25	*Chenopodium album*.		3.14	5.21			2.28	2.17		
26	*Agropyron cristatum*								1.61	
27	*Artemisia verbenacea*								1.61	
28	*Artemisia gmelinii*				10.65		8.12			3.07
29	*Artemisia ordosica*					11.3	5.17	12.37		7.42
30	*Cirsium arvense* var. *integrifolium*						9.76	14.98		
31	*Ixeris chinensis*		2.71					2.3		
32	*Setaria viridis*							7.23		
33	*Erodium stephanianum*							2.21		6.38
34	*Cynanchum thesioides* (Freyn) K.Schum.							2.17		
35	*Ixeris sonchifolia* (Bunge) Hance							2.13		
36	*Linaria vulgaris* subsp. *Chinensis*					4.94				
37	*Taraxacum mongolicum*					5.71				
38	*Euphorbia esula* L.					2.42				3.07
39	*Tribulus terrestris* L.					10.88				
40	*Cynanchum chinense* R. Br.						2.24			
41	*Rhaponticum uniflorum* (L.) DC.					2.41				
42	*Saussurea japonica*						2.24			
43	*Sonchus arvensis* L.						2.24			
44	*Lespedeza potaninii*			2.6						
45	*Amorpha fruticosa* L.			2.6						
46	*Eragrostis pilosa*			17.75	6.08					10.62
47	*Artemisia indica*		2.71							
48	*Primula farinosa* L.		2.71							
49	*Melandrium apricum*.		2.71							
50	*Amaranthus viridis* L.		2.85							
51	*Pharbitis purpurea* (L.) Voigt			2.6						
52	*Chloris virgata*		3.14		11.71					
53	*Medicago falcata* L.		2.71							
54	*Echinops gmelinii* Turcz.				8.1					
55	*Krascheninnikovia ceratoides*				2.11					
56	*Setaria pumila*				2.11					
57	*Astragalus melilotoides* Pall.					5.04				
58	*Chenopodum aristatum* L.					4.94				
59	*Artemisia capillaris*								3.53	
60	*Lappula myosotis*				2.11					

In the table, “S” represents a sunny slope and “N” represents a shady slope.

#### 3.2.2 Analysis of the importance value of platform vegetation in tailings dumps with different restoration durations

Analysis of the distribution in **Table 2** indicates that *Medicago sativa* is present across landfill platforms with four different restoration durations, although its importance value gradually declines as the recovery period increases. In the early stages of restoration, *Medicago sativa* exhibits the highest importance value, followed by *Imperata cylindrica* and *Salsola collina*. As restoration progresses, native geomorphological species gradually invade and predominate: by the 6-year mark, first- and second-year native plants hold relatively high importance values, with a small presence of perennial species also emerging; by 12 years, the importance values of perennial plants such as *Leymus chinensis* and *Aster altaicus* have significantly increased, leading to changes in the community structure as invasive species begin to penetrate the *Medicago sativa* assemblage and secure a certain ecological position. Moreover, with extended restoration periods, species such as *Imperata cylindrica* and Chenopodium gradually vanish, while species like *Microstegium vimineum* and *Artemisia ordosica* begin to appear. The calculation of the important value refers to the Equation 1 in 2.2.1.

**Table 2:** Plant importance values on the spoil heap platform under different restoration years.

Number	Plant name	Important value%			
		2Y	6Y	10Y	12Y
1	*Medicago sativa L.*	49.31	32.26	15.04	11.22
2	*Salsola collina Pall.*	20.68		22.18	
3	*Imperata cylindrica (L.) Beauv.*	15.36			
4	*Artemisia sieversiana*	9.25			8.55
5	*Chenopodium album L.*	5.40	5.40		
6	*Grubovia dasyphylla*		20.36	10.40	4.51
7	*Chloris virgata*		12.11		
8	*Eragrostis pilosa*		6.87		
9	*Artemisia scoparia Waldst. et Kit.*		6.50		3.97
10	*Pennisetum centrasiaticum Tzvel.*		5.86		3.90
11	*Aster altaicus.*		5.47	9.28	11.07
12	*Artemisia annua L.*		5.17	8.28	8.13
13	*Artemisia ordosica Krasch.*			7.80	9.33
14	*Leymus secalinus (Georgi) Tzvel.*			13.07	8.85
15	*Taraxacum mongolicum Hand.-Mazz.*			7.65	
16	*Euphorbia esula L.*			6.30	
17	*Leymus chinensis (Trin.) Tzvel.*				15.50
18	*Bidens parviflora Willd.*				10.49
19	*Conyza canadensis (L.) Cronq.*				4.48

### 3.3 Analysis of slope vegetation diversity in tailings dumps with different restoration durations

**Figure 3:**
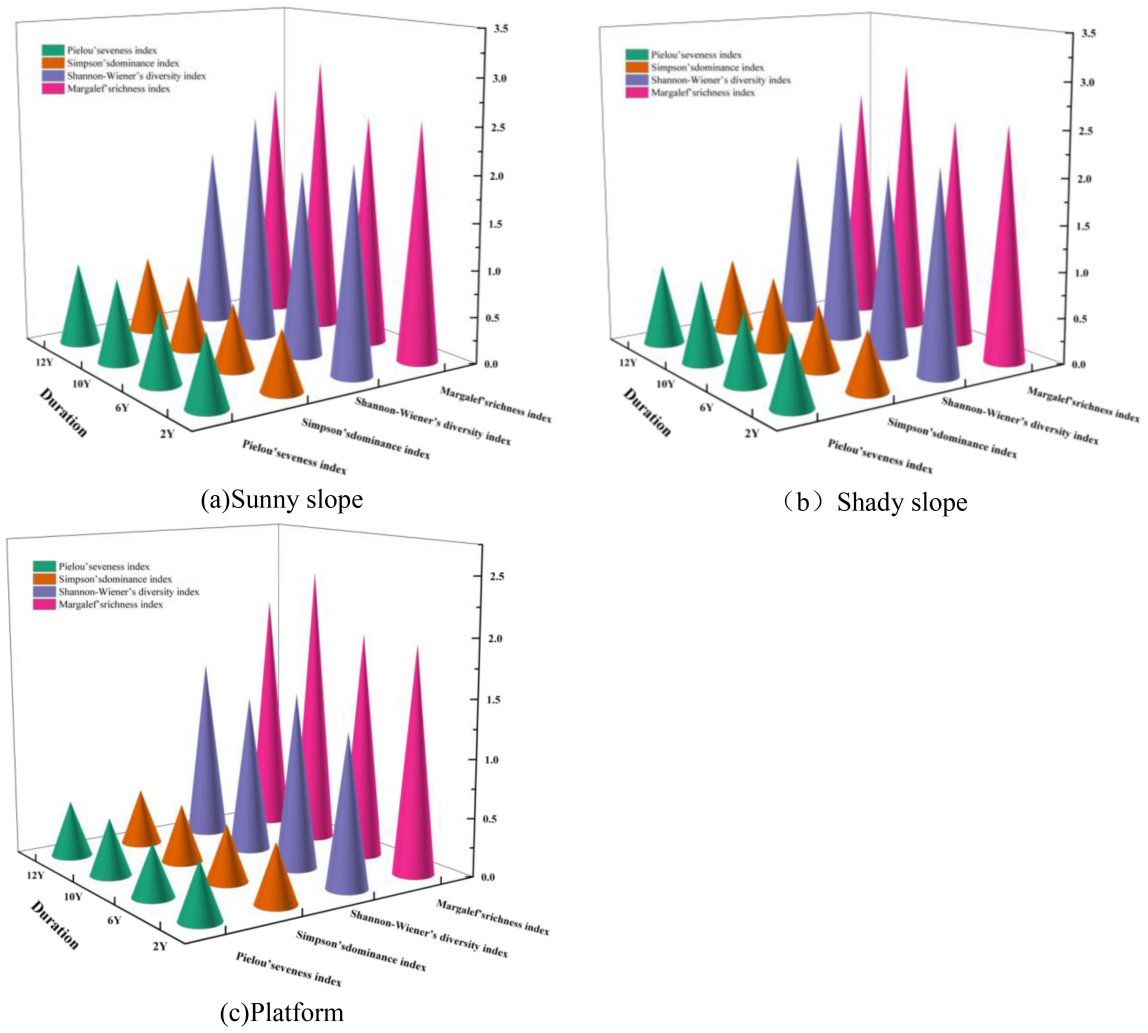
Plant diversity on the spoil heap under different restoration durations. **(a)** The diversity of plants on the sunny slope of the dump; **(b)** Plant diversity on the shady slope of the dump; **(c)** The diversity of plant community characteristics of dump platform.

The stability of community functions can be reflected in species diversity. According to the data in (**Figures 3a, b**), the species diversity index is relatively high during the early stages of restoration. Although there are overall fluctuations, the amplitude of these fluctuations is relatively small. Specifically, both the Margalef richness index and the Shannon-Wiener diversity index show an upward trend at 10 years of restoration, followed by a slight decline at 12 years. In contrast, the Pielou evenness index and the Simpson dominance index exhibit similar trends; both indices fluctuate at 10 years, with this fluctuation persisting until the end of the 12-year period, and overall, they display a gradual increase with the extension of the restoration period. A comparison of the indices for species on different slope aspects at the same restoration age reveals that the Shannon-Wiener diversity index is generally higher on the shady slopes than on the sunny slopes. The Margalef richness index is higher on sunny slopes than on shady slopes during the early stages of restoration, but no significant difference is observed in the later stages. The Simpson dominance index is higher on sunny slopes than on shady slopes during the later stages of restoration, while the Pielou evenness index does not show a significant difference between the different slope aspects.

According to the data in (**Figure 3c**), the species diversity index is at a relatively low level during the early stages of restoration, with an overall small amplitude of fluctuation. Specifically, the Margalef richness index has the highest values among all indices and shows a gradual increase with the extension of the restoration period. The Shannon-Wiener diversity index exhibits a phased characteristic, declining at 10 years and then rebounding at 12 years, while both the Pielou evenness index and the Simpson dominance index maintain relatively low values, with trends that gradually increase as the restoration period extends. The calculation of the important value refers to the Equation 1 in 2.2.1.

### 3.4 Soil C, N, and P content in tailings dumps with different restoration durations

**Figure 4:**
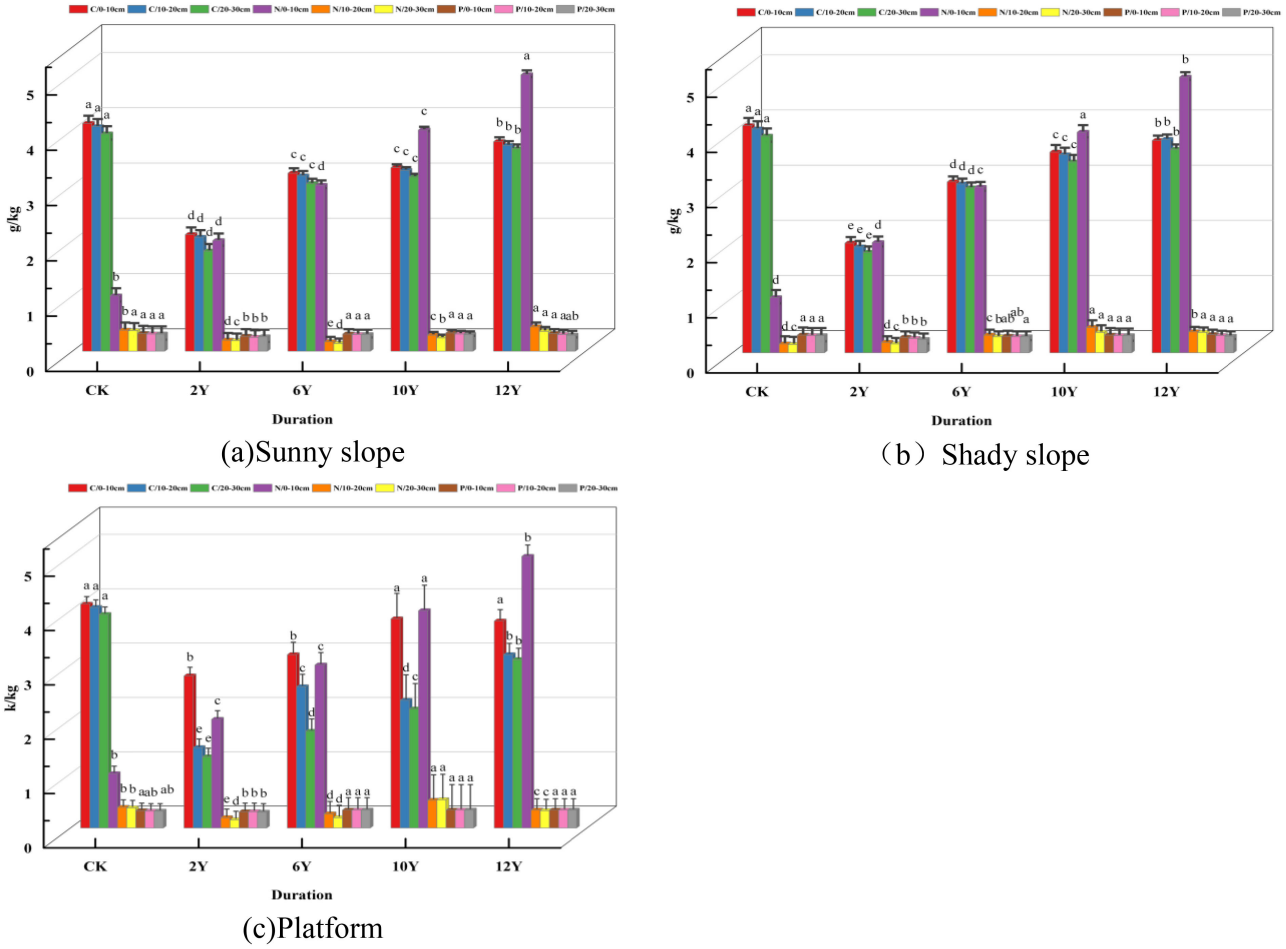
Soil C, N, and P Contents at the Dumping Site under Different Restoration Periods. **(a)** The content of soil C, N and P in the sunny slope of the dump; **(b)** The content of C, N and P in the shady slope soil of the dump; **(c)** The contents of C, N and P in the soil of the dump platform. Different lowercase letters indicated that there were significant differences in C, N and P among different years (P < 0.05).

As shown in (**Figure 4a**), the organic carbon content showed a significant upward trend with the increase of recovery years. The carbon content of CK (unexploited original landform) was the highest (0-10cm: 4.12 g/kg), and the lowest in the early stage of recovery (2Y) (0-10cm: 2.11 g/kg). At 12Y, the carbon content (3.79 g/kg) was close to the CK level, but still slightly lower. Under each restoration year, the carbon content decreased with the deepening of the soil layer, indicating that vegetation restoration had a stronger effect on surface soil carbon accumulation. For example, 0-10cm (3.79 g/kg) > 20-30cm (3.66 g/kg) in 12Y plot. The nitrogen content showed a ‘ V ‘ trend. The lowest nitrogen content (0.14-0.21 g/kg) was found in 2Y and 6Y plots, which was significantly lower than that of CK (0.37-0.41 g/kg). It began to rise at 10Y (0.24-0.36 g/kg); it peaked at 12Y (0.36-0.44 g/kg) and even slightly exceeded CK. Nitrogen content decreased with the deepening of soil layer, such as 0-10cm (0.44 g/kg) > 20-30cm (0.36 g/kg) in 12Y plot, which was consistent with the trend of carbon distribution. Overall stability: the phosphorus content fluctuated little between different recovery years. There was no significant difference in phosphorus content between CK and 6Y, 10Y, 12Y plots (0.29-0.33 g/kg). Only the phosphorus content of 2Y plot was slightly lower (0.25-0.27 g/kg). The phosphorus content was evenly distributed in the 0–30 cm soil layer, and there was no obvious vertical change, indicating that the mobility of phosphorus was weak and limited by vegetation restoration.

As shown in (**Figure 4b**), the organic carbon content increased significantly with the recovery years. CK (unexploited original landform) had the highest carbon content (0-10cm: 4.12 g/kg), and 2Y plot had the lowest carbon content (0-10cm: 1.99 g/kg). The carbon content (3.85 g/kg) in the 12Y plot was close to the CK level. Under different restoration years, the carbon content decreased with the deepening of soil layer, and the carbon content of 0-10cm (3.85 g/kg) > 20-30cm (3.69 g/kg) in 12Y plot, indicating that vegetation restoration had obvious effect on surface soil carbon accumulation. The nitrogen content increased first and then decreased. The nitrogen content of 2Y (0.16-0.23 g/kg) was similar to that of CK (0.15-0.18 g/kg), 10Y reached the peak (0.37-0.51 g/kg), and 12Y decreased slightly (0.37-0.41 g/kg). Nitrogen content decreased with the deepening of soil layer, such as 0-10cm (0.51 g/kg) > 20-30cm (0.37 g/kg) in 10Y plot, which was consistent with the trend of carbon distribution. The change of phosphorus content is small. The calculation of Margalef richness index, Shannon-Wiener diversity index, Pielou evenness index and Simpson dominance index refer to the Equations 2–5 in 2.2.1. It was slightly lower in plot 2Y (0.24-0.28 g/kg), and there was no significant difference between plot 6Y (0.29-0.33 g/kg) and CK (0.31-0.32 g/kg). The difference of phosphorus content in each soil layer was not significant, indicating that the vertical migration of phosphorus was weak.

As shown in (**Figure 4c**), with the increase of restoration years, soil organic carbon content showed a significant recovery trend. In the 0–10 cm surface soil, the organic carbon content of CK (unexploited primitive landform) was the highest (4.12 g/kg), while it was significantly reduced to 2.8 g/kg at the initial stage of restoration (2Y). With the extension of the recovery period, the organic carbon content gradually approached the CK level, reaching 3.81 g/kg at 12Y, which was not significantly different from CK (p > 0.05). Under each recovery period, the organic carbon content decreased with the increase of soil depth. For example, at 12Y, the contents of 0-10cm, 10-20cm and 20-30cm soil layers were 3.81,3.19 and 3.1g/kg, respectively. The total nitrogen content showed a trend of decreasing first, then increasing and then stabilizing. The lowest nitrogen content (0.15-0.23 g/kg) was found in the plot of 2Y, which was significantly lower than that of CK (0.37-0.41 g/kg). It reached the peak value (0.51-0.57 g/kg) at 10Y, which may be related to the rapid growth of vegetation and the enhancement of nitrogen fixation at this stage. A slight decrease (0.32-0.39 g/kg) was observed at 12Y, but it was still significantly higher than the initial (2Y). The nitrogen content decreased with the increase of soil depth, which was consistent with the distribution of organic carbon. For example, the nitrogen content of 0–10 cm (0.39 g/kg) was significantly higher than that of 20–30 cm (0.32 g/kg) in 12Y plot. The total phosphorus content fluctuated little between different restoration years and soil layers. The phosphorus content of CK and recovery plots (6Y, 10Y, 12Y) remained in the range of 0.31-0.33 g/kg, and there was no significant difference (p > 0.05). Only the phosphorus content of 2Y plot was slightly lower (0.29-0.3 g/kg), but the difference was not significant. The phosphorus content was evenly distributed in the 0–30 cm soil layer and did not show obvious vertical variation.

### 3.5 Characteristics of soil stoichiometric ratios in tailings dumps with different restoration durations

**Figure 5:**
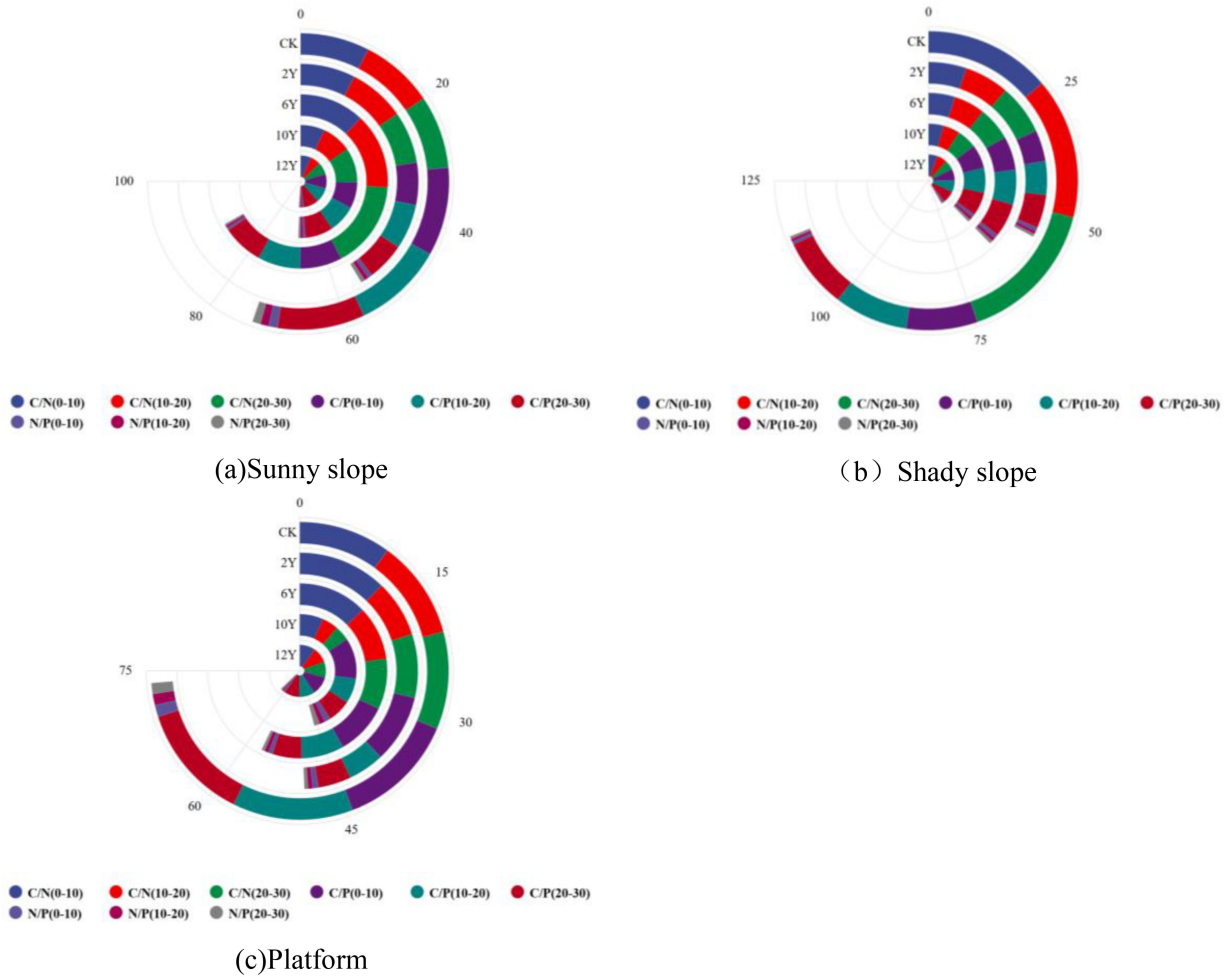
Characteristics of soil stoichiometric ratio of dump platform with different restoration years. **(a)** is the soil stoichiometric ratio of the sunny slope of the dump; **(b)** is the soil stoichiometric ratio of the shady slope of the dump; **(c)** is the soil stoichiometric ratio of the dump platform.

As shown in (**Figure 5a**), the C/N ratio showed a trend of increasing first and then decreasing. The 6Y plot reached the peak (16.10-21.64), which was significantly higher than that of CK (10.05-10.68), indicating that the soil carbon accumulation rate exceeded the nitrogen accumulation. The C/N ratio (8.45-10.17) in the 12Y plot has returned to close to or below the CK level. Under different restoration years, the C/N ratio generally increased with the deepening of soil layer, and the C/N ratio (21.64) of 20–30 cm soil layer in 6Y plot was significantly higher than that of surface layer (16.10). The C/P ratio showed a gradual recovery trend. The lowest (6.96-8.24) was found in 2Y, which was significantly lower than CK (12.68-13.10). With the increase of restoration years, the C/P ratio gradually increased, and the 12Y plot (11.84-12.62) was close to the CK level. The difference of C/P ratio among different soil layers was small, and there was no obvious vertical variation. The N/P ratio showed a ‘ V ‘ type change. The 6Y plot reached the lowest value (0.47-0.65), which was significantly lower than CK (1.19-1.28). The N/P ratio (1.24-1.47) in the 12Y plot has returned to the CK level or slightly higher. The difference of N/P ratio in the vertical direction was small, but the 6Y and 10Y plots showed a decreasing trend with the deepening of soil layer.

As shown in (**Figure 5b**), compared with CK (22.89-26.20), the C/N ratio of each restoration year was significantly reduced (7.14-11.38). During the recovery process, the C/N ratio showed a trend of decreasing first and then increasing, 10Y reached the lowest value (7.14-9.35), and 12Y rebounded slightly (9.39-10.18). Under each recovery period, the C/N ratio increased with the deepening of soil layer, which was consistent with CK, but the absolute value was significantly lower than CK. The C/P ratio showed a continuous upward trend. The lowest (7.11-7.65) was found in 2Y plot, which was significantly lower than CK (12.68-13.29). With the increase of restoration years, the C/P ratio (11.67-12.42) of 12Y plot was close to CK level. The ratio of C/P changed little among different soil layers, and did not show obvious vertical gradient. The N/P ratio was significantly higher than that of CK (0.48-0.56), and showed a change of first increase and then decrease. 10Y reached the peak (1.19-1.59), 12Y decreased slightly (1.23-1.24), but still maintained at a high level. The N/P ratio decreased with the deepening of soil layer, and the recovery years were consistent.

As shown in (**Figure 5c**), the C/N ratio shows significant fluctuations. The 10Y plot reached the lowest value (4.21-6.75), which was significantly lower than CK (10.05-10.68). The 12Y plot (9.67-9.77) has recovered to near CK level. Except for 2Y plot, the C/N ratio of each restoration year did not change much with the deepening of soil layer, which was different from the vertical distribution pattern of CK. The C/P ratio showed an overall upward trend. The 2Y plot was the lowest (4.52-9.33), and the 12Y plot (9.39-11.55) was close to CK (12.68-13.10). In the early recovery stage (2Y, 6Y), the C/P ratio decreased significantly with the deepening of soil layer, and the vertical difference decreased in the later stage (10Y, 12Y). The N/P ratio fluctuated greatly. The peak value (1.55-1.73) of 10Y plot was significantly higher than that of CK (1.19-1.28). The 12Y plot (0.97-1.18) fell back to the CK level. Except for the 10Y plot, the N/P ratio generally decreased with the deepening of the soil layer, which was consistent with the CK trend.

### 3.6 Relationship between vegetation communities and soil stoichiometric ratios in tailings dumps with different restoration durations

#### 3.6.1 Principal component analysis of vegetation diversity and soil stoichiometric ratios in tailings dumps

**Figure 6:**
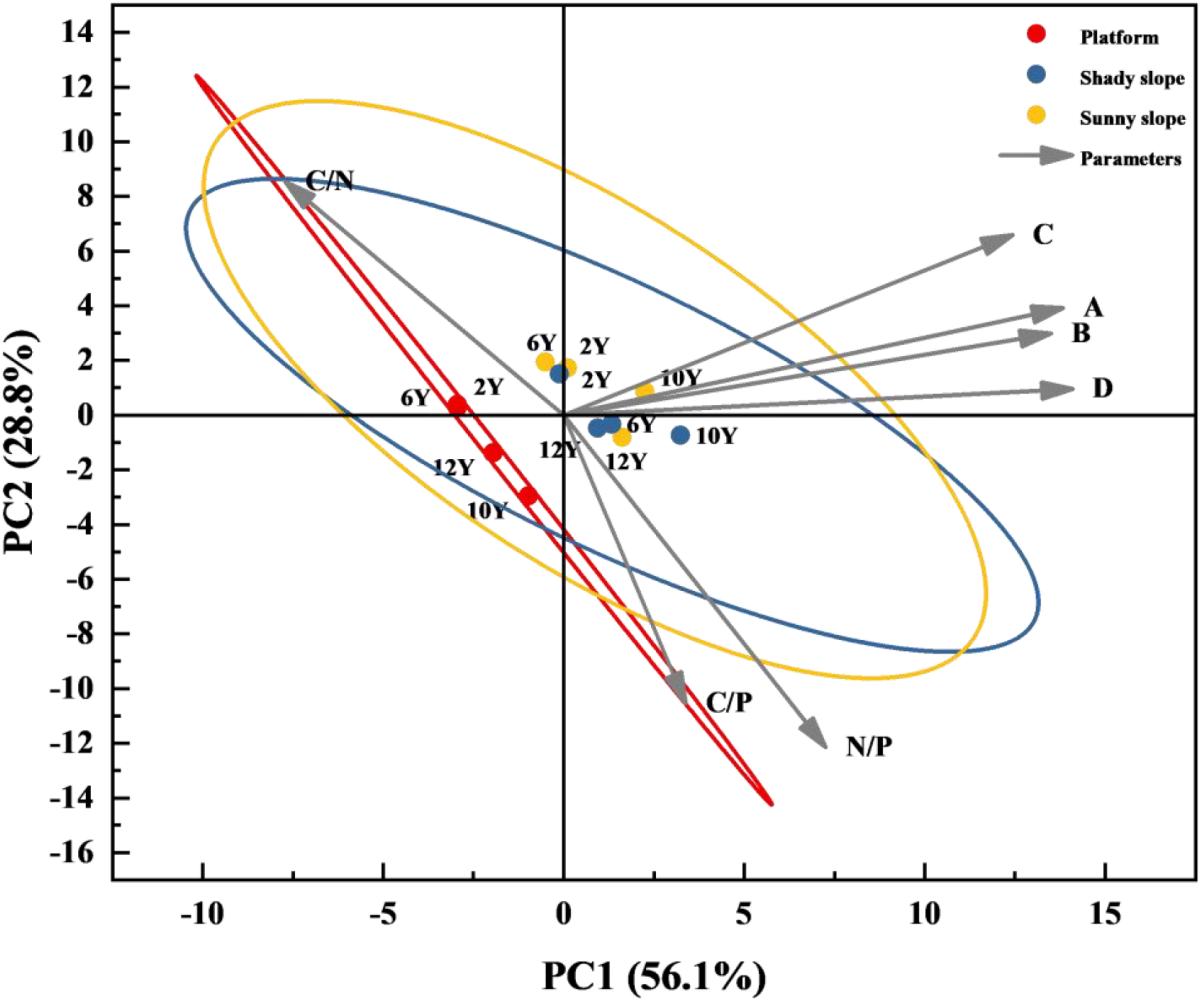
Principal component analysis of vegetation diversity and soil stoichiometric ratio in dump. **(A)** is Pielou ‘seveness index; **(B)** is Simpson ‘sdominance index; **(C)** is Shannon-Wiener ‘s diversity index; **(D)** is Margalef ‘srichness index.

It can be seen from **Figure 6** that in order to quantify the correlation strength between vegetation diversity index and soil stoichiometric ratio, the load value and contribution of each parameter on the principal component axis are clarified. Combined with the results of two-way ANOVA, the ecological significance of principal components was further analyzed. The larger the absolute value of the load value (usually | load value | ≥ 0.5 is regarded as a strong correlation), the closer the correlation between the parameter and the corresponding principal component; the contribution rate of principal component reflects the ability of the axis to explain the total variation, and the cumulative contribution rate ≥ 75% can explain most of the ecological process variation.

PC1 (contribution rate 56.1%) was the vegetation-soil collaborative restoration axis. On the PC1 axis, the positive high-load parameters concentratedly reflected the ‘ collaborative optimization in the restoration process ‘: among the vegetation diversity indexes, the Margalef richness index (R = 0.81) and Shannon-Wiener diversity index (H = 0.75) had the highest loads, indicating that with the increase of restoration years, the number of species increased (such as 60 species on the 12Y slope) and the community structure was more complex, which was consistent with the original conclusion that the species composition tended to be diverse and the native plants were dominant. In the soil stoichiometric ratio, N/P (0.83) and C/P (0.78) loads were significant. N/P (1.24-1.47) and C/P (11.84-12.62) in the 12Y plot were close to CK (N/P 1.19-1.28, C/P 12.68-13.10), indicating that the soil nitrogen and phosphorus balance, carbon and phosphorus cycle gradually recovered, and nutrient limitation (especially nitrogen limitation) was significantly alleviated. The gradient distribution of PC1 axis (2Y plots are concentrated in the negative area of PC1, and 12Y plots are concentrated in the positive area) directly reflects the core process of ‘ restoration years driving the transition of vegetation-soil system from ‘ degradation imbalance ‘ to ‘ near-natural stability ‘.

PC2 (contribution rate 22.3%) was the micro-topographic nutrient differentiation axis. The high load parameter of the PC2 axis is C/N (-0.68), and the topographic difference has a significant effect on the distribution of the axis: the PC2 score of the shady slope is generally lower than that of the sunny slope, because the C/N vertical consistency of the shady slope is strong (the C/N correlation coefficient of each soil layer is r > 0.8), the organic matter decomposition rate is stable, and the C/N fluctuation is small (12Y shady slope C/N 9.39-10.18); the PC2 score of the platform plot was concentrated, and the C/N ratio was maintained at a low level due to manual management (sprinkler irrigation, mowing) (12Y platform C/N 9.67-9.77), and the carbon-nitrogen-carbon-phosphorus co-accumulation (C/N and C/P were significantly positively correlated), which was compared with the C/N differentiation of the slope. The variation of PC2 axis was mainly due to the difference of soil carbon and nitrogen cycle caused by micro-topography (sunny slope/shady slope/platform), which explained the internal mechanism of the difference of recovery effect of different topography in the same year.

#### 3.6.2 Correlation analysis of soil stoichiometric ratios at different soil depths in tailings dumps

**Figure 7:**
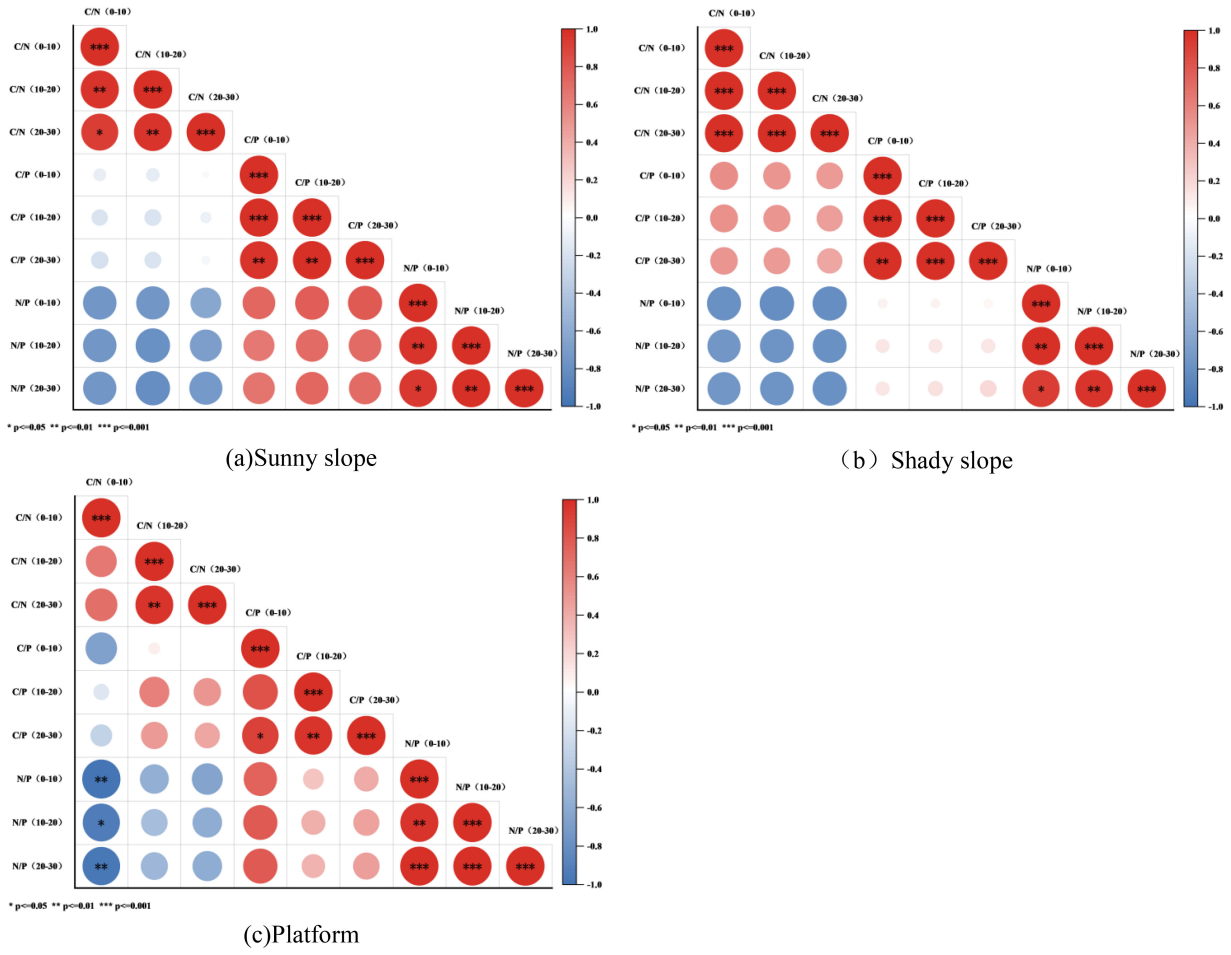
Correlation Analysis of Soil Stoichiometric Ratios at Different Soil Depths in the Waste Disposal Site. **(a)** Correlation analysis of soil stoichiometric ratio at different soil depths on the sunny slope of the dump; **(b)** Correlation analysis of soil stoichiometric ratio at different soil depths on the shady slope of the dump; **(c)** Correlation analysis of soil stoichiometric ratio in different soil depths of dump platform. *** is extremely significant correlation, ** is significant correlation, * is correlation.

As shown in (**Figure 7a**), the C/N ratio of the 0–10 cm soil layer is extremely significantly positively correlated with that of the 10–20 cm soil layer (***), indicating a strong synchronicity in the variation of carbon-to-nitrogen ratios between the top and middle layers. The C/N ratio of the 10–20 cm layer is also extremely significantly positively correlated with that of the 20–30 cm layer (*), revealing a clear cooperative change in carbon-to-nitrogen ratios between the middle and deeper layers. Similarly, the C/P ratio of the 0–10 cm layer is extremely significantly positively correlated with that of the 10–20 cm layer (***), suggesting a close association in carbon-to-phosphorus ratios between the top and middle layers, while the C/P ratio of the 10–20 cm layer is extremely significantly positively correlated with that of the 20–30 cm layer (*), indicating consistent changes in carbon-to-phosphorus ratios between the middle and deeper layers. In contrast, the N/P ratio of the 0–10 cm layer is extremely significantly negatively correlated with that of the 10–20 cm layer (***), reflecting opposite trends in nitrogen-to-phosphorus ratios between the top and middle layers, and the N/P ratio of the 10–20 cm layer is extremely significantly negatively correlated with that of the 20–30 cm layer (*), showing an inverse relationship in nitrogen-to-phosphorus ratios between the middle and deeper layers. Moreover, no significant correlation was observed between the C/N and C/P ratios within the individual soil layers, suggesting that the regulatory mechanisms of carbon-to-nitrogen and carbon-to-phosphorus ratios are relatively independent. Additionally, a significant negative correlation between the C/N and N/P ratios was found in some layers (e.g., between the 0–10 cm C/N and the 20–30 cm N/P ratios, *), reflecting an antagonistic relationship between carbon–nitrogen balance and nitrogen–phosphorus balance.

According to (**Figure 7b**), the C/N ratios between the soil layers exhibit a highly significant positive correlation (*), with correlation coefficients (greater than 0.8) that are higher than those on sunny slopes. This suggests that the vertical consistency of soil carbon-nitrogen ratios is stronger on shaded slopes, which may be related to stable moisture conditions and uniform organic matter decomposition. The C/P ratios between the 0–10 cm and 10–20 cm layers, as well as between the 10–20 cm and 20–30 cm layers, are highly significantly positively correlated; the vertical associations are similar to those on sunny slopes, albeit with slightly higher correlation values. In contrast, the N/P ratios between the 0–10 cm and 10–20 cm layers and between the 10–20 cm and 20–30 cm layers are highly significantly negatively correlated, with a stronger negative correlation than that observed on sunny slopes, indicating more pronounced vertical differentiation of soil nitrogen-phosphorus ratios on shaded slopes. Moreover, in certain soil layers (e.g., the 0–10 cm C/P and 20–30 cm N/P), the C/P and N/P ratios are significantly negatively correlated, with a stronger negative relationship than that on sunny slopes, reflecting a tighter coupling between soil carbon-phosphorus and nitrogen-phosphorus ratios on shaded slopes.

According to (**Figure 7c**), the C/N ratios of the 0–10 cm and 10–20 cm soil layers exhibited an extremely highly significant positive correlation (*), and the C/N ratios of the 10–20 cm and 20–30 cm layers also showed an extremely significant positive correlation. The vertical consistency lies between that of the sunny and shady slopes, which may be attributed to the relatively thick platform topsoil (approximately 2 meters) and its more stable soil structure. The C/P ratios of the 0–10 cm and 10–20 cm layers displayed an extremely highly significant positive correlation, whereas the correlation between the 10–20 cm and 20–30 cm layers was weaker, indicating that the stability of the deep soil carbon-to-phosphorus ratio on the platform is relatively low, possibly due to the influence of underlying waste material. The N/P ratios among the different soil layers were significantly negatively correlated, with the 0–10 cm and 10–20 cm layers exhibiting an extremely highly significant negative correlation (*), the strength of which is lower than that of the shady slope but higher than that of the sunny slope. Additionally, on the platform, the C/N and C/P ratios were significantly positively correlated in some soil layers (e.g., between the 0–10 cm C/N and the 0–10 cm C/P, ***), differing from the nonsignificant associations observed on the sunny and shady slopes. This suggests a synergistic effect in the accumulation processes of soil carbon-nitrogen and carbon-phosphorus on the platform, potentially related to mowing management and sprinkler irrigation practices associated with the artificial cultivation of Medicago sativa.

## 4 Discussion

### 4.1 Driving mechanism of restoration years on soil organic carbon accumulation in dump-based on ecological stoichiometric carbon cycle theory

In this study, through long-term monitoring of the dump slope and platform of Manlailiang Open-pit Coal Mine, it was found that with the increase of ecological restoration years, the soil organic carbon content increased significantly. When it was restored to the 12^th^ year, the surface soil organic carbon was close to the level of unmined original landform (CK), especially the surface carbon accumulation effect was obvious. This change reflects the dynamic regulation process of carbon input and decomposition balance in vegetation-soil system, which is consistent with the research conclusion of Yang et al. (2024) in grassland open-pit mine, that is, the return of vegetation litter and root exudates are the main sources of soil organic carbon increase.

The analysis based on ecological stoichiometry theory showed that the microbial decomposition efficiency was low due to the low coverage of Medicago sativa, less litter and high C/N ratio (15-25) in the early stage of restoration (2Y), resulting in insufficient soil organic carbon input (only 2.11 g/kg in 0–10 cm soil layer). With the recovery process, native perennial plants such as Aster tataricus and Artemisia ordosica gradually became dominant, and their litter C/N ratio was low (10-20), which was more easily decomposed by microorganisms and converted into soil organic carbon. In addition, the deep root exudates continuously input carbon sources, so that the surface soil organic carbon increased to 3.79-3.85 g/kg in the 12th year, which was close to 4.12 g/kg of CK.

Lei et al. (2023) proposed that there was a "threshold effect" in the recovery of soil organic carbon in the northern open-pit mine area, that is, the growth rate of carbon pool slowed down and tended to the natural level after 10–15 years. The 12-year monitoring results of this study showed that there was no significant difference in organic carbon content between CK and CK (p > 0.05), confirming the effect. However, different from the conclusion of Lei Shaogang et al., "platform carbon accumulation is faster than slope", the organic carbon content of slope and platform in this study is similar (slope 3.79-3.85 g/kg, platform 3.81 g/kg). The reason is that Manlailiang mining area has adopted targeted management measures: setting sand barrier on the slope to reduce carbon loss caused by soil erosion; although the platform removed some litter due to cutting alfalfa, it promoted vegetation growth through artificial sprinkler irrigation and compensated for carbon input. These measures effectively promoted the simultaneous recovery of carbon pools in different terrains, indicating that artificial management can alleviate topographic constraints by regulating the carbon "input-loss" process, which is consistent with the view proposed by (Zhang et al., 2023) that "near-natural vegetation configuration combined with artificial regulation can optimize the carbon cycle".

### 4.2 Coupling mechanism of soil stoichiometric ratio evolution and vegetation succession-combined with nutrient limitation and growth rate hypothesis

#### 4.2.1 C/N ratio: strategies for regulating organic matter decomposition and vegetation nutrient utilization

The results of this study showed that the soil C/N ratio on the sunny slope showed a trend of ‘ first increase and then decrease ‘ (peaked at 16.10-21.64 in 6 years, and fell back to 8.45-10.17 in 12 years), while the C/N vertical consistency of each soil layer on the shady slope was strong (correlation coefficient of each layer > 0.8). This difference can be explained by the nutrient limitation theory and the "plant ecological stoichiometric indication hypothesis" proposed by (Tian et al., 2021).

Specifically, the evapotranspiration of the sunny slope was strong and the water was insufficient. At 6 years, the vegetation carbon input rate exceeded the nitrogen release (microbial decomposition was limited by water), resulting in an increase in C/N. At this time, soil nitrogen supply is insufficient, and plants adapt to the environment by improving nitrogen use efficiency (such as increasing root absorption). To 12 years, with the planting of native plants such as Leymus chinensis, the litter nitrogen content increased (Gramineae total nitrogen 1.5% -2.5%), and the improvement of vegetation coverage promoted microbial activity and nitrogen mineralization, so that C/N fell back to close to CK level, nitrogen limitation was alleviated, and the plant strategy changed from "nutrient efficient utilization" to "biomass accumulation", which was in line with the expectation of low C/N to promote growth in the growth rate hypothesis.

Due to the stability of water, the microbial decomposition rate of the shady slope was small in the vertical direction, and the release of carbon and nitrogen was synchronized. The C/N of each layer was significantly positively correlated and was always lower than that of the sunny slope in the same period. This indicates that plants on the shady slope do not need to over-adapt to nitrogen limitation, so their growth status is better (the importance value of perennial plants on the shady slope is 54.56% in 12 years, and the biomass is higher than that on the sunny slope). The results further support the conclusion of (Wang et al., 2024b) that "water-stoichiometry synergistically regulates vegetation growth in semi-arid areas".

#### 4.2.2 C/P and N/P ratio: Indicating phosphorus limitation dynamics and plant functional type transformation.

This study showed that with the increase of restoration years, the soil C/P ratio continued to rise (from 6.96-8.24 in 2Y to 12.68-13.10 in 12Y), while the N/P ratio showed a "V" type change (the lowest was 0.47-0.65 in 6Y, and increased to 1.24-1.47 in 12Y). Based on the nutrient limitation theory (N/P20 is phosphorus limitation) and (Ai et al., 2025) ’s research on "soil stoichiometric ratio driving plant nutrient uptake strategy", the nutrient limitation stages in the process of vegetation succession can be divided as follows:

In the early stage of recovery (2Y, 6Y): N/P was lower than 1, indicating that nitrogen was extremely deficient. At this stage, alfalfa (Leguminosae nitrogen-fixing plant) became the dominant species (important value 26.06% -18.17%) by virtue of nitrogen fixation ability, and its nitrogen fixation gradually replenished soil nitrogen, laying a foundation for subsequent succession, which was consistent with the conclusion of (Shan et al., 2024) that "nitrogen-fixing plants in the early stage of mining wasteland give priority to improving the nitrogen environment".

In the middle of recovery (10Y): N/P increased to 0.8-1.2, and nitrogen limitation was alleviated. At this time, the importance value of one-year-old and two-year-old plants (such as Setaria viridis) increased due to the high growth rate, which was consistent with the growth rate hypothesis and similar to the phenomenon of "mid-term fast-growing plant dominance" observed by (Liu et al., 2024) in the alpine region.

At the late stage of recovery (12Y): N/P reached 1.24-1.47, which was similar to CK (1.19-1.28), indicating that the balance of nitrogen and phosphorus was basically established. Perennial protophytes (such as Artemisia ordosica and Leymus chinensis) have become dominant species, with low growth rate but high phosphorus utilization efficiency (insoluble phosphorus is dissolved by organic acids secreted by roots), and can maintain phosphorus cycle through litter return to adapt to stable nutrient environment, which verifies the law of (Wang, 2025) "soil stoichiometric balance drives plant functional type transformation".

In addition, the soil phosphorus content was generally stable (0.29-0.33 g/kg) and the distribution of each soil layer was uniform, indicating that phosphorus was mainly controlled by parent material and less affected by vegetation, which was consistent with the conclusion of (Yuan et al., 2025) that phosphorus in southwest coal gangue yard was not sensitive to vegetation restoration. The change of C/P was mainly driven by the fluctuation of carbon content, which was compared with that of N/P driven by nitrogen.

### 4.3 Regulation of nitrogen cycle by nitrogen-fixing species (*Medicago sativa*) and promotion of native species colonization

As an artificially planted nitrogen-fixing plant, *Medicago sativa* plays an important "nitrogen foundation" role in the early stage of ecological restoration (2Y, 6Y) of the dump. It is symbiotic with rhizobium, and the annual nitrogen fixation amount reaches 150–200 kg/hm2, which significantly alleviates the serious shortage of soil nitrogen in the early stage (2Y soil nitrogen content is only 0.14-0.21 g/kg), which is consistent with the conclusion of Li Shimei (Tian et al., 2021) that "Legume nitrogen fixation is positively correlated with soil nitrogen accumulation".

From the perspective of ecological stoichiometry, the nitrogen fixation of *Medicago sativ*a not only directly increased soil nitrogen content, but also enhanced microbial activity by reducing soil C/N ratio (from 12–15 in 2Y to 10–12 in 6Y). The lower C/N ratio weakened the competition between microorganisms and plants for nitrogen, promoted the mineralization of organic matter and the release of available nitrogen, and formed a positive feedback cycle of "nitrogen fixation-microbial mineralization-nitrogen release", which laid a nutrient foundation for the colonization of native species.

With the recovery process, the important value of *Medicago sativ*a decreased from 26.06% of 2Y to 6.62% of 12Y. This change reflects its indirect promotion to local species: on the one hand, *Medicago sativa* improves the soil nitrogen environment and creates suitable conditions for local species such as Aster tataricus and Leymus chinensis, which is consistent with the phenomenon observed by (Zhao et al., 2023) that "nitrogen-fixing plants pave the way for local species"; on the other hand, the humus formed by litter decomposition improved the soil structure, enhanced the ability of water and fertilizer conservation, and reduced the difficulty of local planting. In addition, *Medicago sativa* inhibits exotic species through niche complementarity, provides support for the competitive advantage of native species, and promotes the transformation of the community from artificial monoculture to native diversity, reflecting the “founder effect” of nitrogen-fixing species. The results also confirm the suggestion of (Jiao et al., 2020) that the ecological function of pioneer species should be paid attention to in the open-pit mine dump in the Yellow River basin.

## 5 Conclusion

(1) Ecological restoration presents three stages of progressive characteristics:

Initial stage (2–6 years): mainly artificial alfalfa, less species (slope < 30 species, platform < 10 species); soil organic carbon ≤ 2.5 g/kg, total nitrogen ≤ 0.21 g/kg, C/N and C/P imbalance, the system only achieve basic coverage, low stability.

In the middle stage (10 years): the native perennial plants such as Aster tataricus and Leymus chinensis in Altay gradually dominated, and the number of species increased to more than 30. Soil organic carbon ≥ 3.5 g/kg, total nitrogen ≥ 0.36 g/kg, nitrogen and phosphorus restrictions eased, and the system entered the stage of "vegetation succession-nutrient optimization".

In the late stage (12 years): the plant community and soil index were close to the natural state (CK), the species of slope and platform were 60 and 19, respectively, and the importance value of native plants was more than 50%; the organic carbon (3.79-3.85 g/kg) and total nitrogen (0.39-0.44 g/kg) were close to the CK level, and the C/N, C/P and N/P recovered to more than 90% of CK. The system achieved "structural stability-functional improvement".

(2) Micro-topography significantly affects the recovery effect:

The water and heat conditions of the shady slope were stable and the recovery was the best: the species diversity was 15% -20% higher than that of the sunny slope, the vertical correlation of soil C/N was strong (r > 0.8), and the decomposition of organic matter and nutrient cycling were more coordinated.

The platform formed a "carbon-nitrogen-carbon-phosphorus synergistic accumulation" mode under sprinkler irrigation and mowing management, and the nutrient supply was stable, but the succession rate of primary plants was slightly lower than that of the shady slope.

## 6 Management advice

1. The restoration strategy was formulated in stages: in the early stage (0-5Y), the alfalfa + salix sand barrier was mainly used to quickly construct the basic vegetation; in the middle stage (5-10Y), the original perennial plants were replanted to accelerate natural succession. In the later stage (10Y +), the intervention was reduced and the stability was maintained by self-regulation of the system.
2. Differential optimization of micro-topography management: adding drip irrigation on sunny slope to improve water and nitrogen mineralization efficiency; the shady slope is preferred as the primary plant planting area; the platform reduces the cutting frequency and retains litter to enhance carbon and nitrogen input.
3. Taking 12 years as the key evaluation node: combined with the proportion of primary plants, soil carbon and nitrogen content and other indicators, it is judged whether the system reaches the “near-natural stable state”, which provides a basis for subsequent management adjustment.

## Data Availability

The original contributions presented in the study are included in the article/supplementary material. Further inquiries can be directed to the corresponding author/s.

## References

[B1] Ai J. Deng J. Wang Y. T. Yang S. L. Zhao Y. Li R. D. . (2025). Soil stoichiometric characteristics and their effects on nutrient driving in sugarcane leaves. Chin. J. Trop. Crops 46, 2458–2468.

[B2] Bi Y. L. Peng S. P. Du S. Z. (2021). Technical difficulties and development direction of ecological reconstruction in open-pit coal mines in arid and semi-arid regions of western China. J. China Coal Soc. 46, 1355–1364. doi: 10.13225/j.cnki.jccs.st21.0707

[B3] Bui E. N. Henderson B. L. (2013). C:N:P stoichiometry in Australian soils with respect to vegetation and environmental factors. Plant Soil 373, 553–568. doi: 10.1007/s11104-013-1823-9

[B4] Cheng B. Zhao Y. J. Zhang W. G. An S. Q. (2010). Research advances in ecological stoichiometry. Acta Ecologica Sin. 30, 1628–1637. doi: 10.20103/j.stxb.2010.06.026

[B5] Cortois R. Schroeder-Georgi T. Weigelt A. van der Putten W. H. De Deyn G. B. (2016). Plant-soil feedbacks: role of plant functional group and plant traits. J. Ecol. 104, 1608–1617. doi: 10.1111/1365-2745.12643

[B6] Deng Y. Y. Zhao T. N. Zhang Y. Shi C. Q. Yue L. L. Chen T. . (2021). Characteristics and source analysis of atmospheric dustfall based on SEM-EDS: A case study of underground coal mining base in desert steppe region. China Environ. Sci. 41, 5512–5521. doi: 10.19674/j.cnki.issn1000-6923.2021.0383

[B7] Fan D. Q. Qiu Y. Sun W. B. Zhao X. S. Mai X. M. Hu Y. W. (2021). Ecological environment assessment of Shenfu mining area based on remote sensing ecological index. Bulletin of Surveying and Mapping 2021 (07), 23–28. doi: 10.13474/j.cnki.11-2246.2021.0203

[B8] Fan Q. C. (2019). Carbon, nitrogen and phosphorus contents and ecological stoichiometric characteristics of *Spartina alterniflora* in the tidal flat wetland of Jiaozhou Bay. Qingdao University, Qingdao. doi: 10.27262/d.cnki.gqdau.2019.002226

[B9] Gou B. W. Wei B. Ma S. M. Nie Y. B. (2020). Distribution characteristics of soil nutrients in the root zone of Haloxylon ammodendron at the southern edge of Gurbantünggüt Desert. Southwest China J. Agric. Sci. 33, 1229–1234. doi: 10.16213/j.cnki.scjas.2020.6.020

[B10] Huang J. Yuan Z. N. (2020). Overview of ecological stoichiometric characteristics and influencing factors of soil carbon, nitrogen, and phosphorus. Modern Agric. Res. 49, 73–76. doi: 10.19704/j.cnki.xdnyyj.2020.01.029

[B11] Ji L. Dong J. H. Fang A. M. Huang Y. L. Li Q. S. Cao Z. G. (2021). Demarcation of sensitive areas and heavy metal accumulation effects in Baorixile large open-pit mine. Chin. J. Ecol. 40, 3325–3338. doi: 10.13292/j.1000-4890.202110.012

[B12] Jiao X. L. Tian J. M. Zhou Y. L. (2020). Ecological restoration evaluation of open-pit mine dump in the Yellow River Basin. Coal Eng. 52, 74–79.

[B13] Lei S. G. Wang W. Z. Li Y. Y. Yang X. C. Zhou Y. L. Duan Y. T. . (2023). Disturbance and restoration of soil organic carbon pool in large open-pit mining areas of northern China. Coal Sci. Technol. 51, 100–109. doi: 10.12438/cst.2023-0965

[B14] Li X. B. Cao Z. W. Zhou J. Huang L. Q. Wang S. F. Yao J. R. . (2019). Mining method transformation and intelligent green mine construction in hard rock mines: A case study of Kaiyang phosphate mine. Chin. J. Nonferrous Metals 29, 2364–2380. doi: 10.19476/j.ysxb.1004.0609.2019.10.18

[B15] Liang X. H. Zhang K. B. Qiao X. (2019). Relationship between soil moisture and nutrients and plant diversity of Caragana microphylla community in semi-arid loess region. Ecol. Environ. Sci. 28, 1748–1756. doi: 10.16258/j.cnki.1674-5906.2019.09.005

[B16] Liu Y. B. Pang J. H. Liang S. Yu D. M. Shi X. P. Li G. R. . (2024). Characteristics of runoff and sediment yield on slopes of mine dump with different vegetation restoration years in alpine region. Bull. Soil Water Conserv. 44, 1–11. doi: 10.13961/j.cnki.stbctb.2024.04.001

[B17] Shandan Cui W. Xing E. D. Yang L. Y. (2024). Impact and evaluation of vegetation restoration on soil nutrients in mining wasteland. J. Shanxi Agric. Univ. (Natural Sci. Edition) 44, 101–110. doi: 10.13842/j.cnki.issn1671-8151.202306032

[B18] Tang C. J. Liu Y. Li Z. W. Guo L. P. Xu A. Z. Zhao J. D. . (2021). Effectiveness of vegetation cover pattern on regulating soil erosion and runoff generation in red soil environment, southern China. Ecol. Indic. 129, 107956. doi: 10.1016/j.ecolind.2021.107956

[B19] Tao Y. Wu G. L. Liu Y. B. . (2017). Soil stoichiometric characteristics and influencing factors of typical shrub communities in the Gurbantünggüt Desert. J. Desert Res. 37, 305–314.

[B20] Tian D. Yan Z. B. Fang J. Y. (2021). Plant ecological stoichiometry characteristics and major hypotheses. Chin. J. Plant Ecol. 45, 682–713. doi: 10.17521/cjpe.2020.0331

[B21] Wang R. R. (2025). Effects of soil carbon, nitrogen, and phosphorus stoichiometry on microbial nutrient utilization strategies in karst forests (Guilin: Guangxi Normal University). doi: 10.27036/d.cnki.ggxsu.2025.002192

[B22] Wang H. Y. Li L. Li Q. Gao X. Y. (2024). Effects of reclamation on soil physical properties of open-pit mine dump in semi-arid area. J. Agric. Sci. Technol. 26, 174–183. doi: 10.13304/j.nykjdb.2023.0548

[B23] Wang S. M. Liu L. Zhu M. B. Shen Y. J. Shi Q. M. Sun Q. . (2024). New ideas for green and low-carbon development of coal under the "Dual Carbon" goal. J. China Coal Soc. 49, 152–171. doi: 10.13225/j.cnki.jccs.YH23.1690

[B24] Wang L. Mu Y. Zhang Q. F. Jia Z. K. (2012). Effects of vegetation restoration on soil physical properties in the wind-water erosion region of the northern Loess Plateau of China. Clean-Soil Air Water 40, 7–15. doi: 10.1002/clen.201100367

[B25] Xu Y. C. Hexigtu Hou Y. S. Jia J. Wang J. W. Li R. B. (2025). Study on ecological environment restoration and mining of open-pit coal mines under green and low-carbon transformation. China Coal 51 (08), 213–220. doi: 10.19880/j.cnki.ccm.2025.08.022

[B26] Xue D. M. Guo X. P. Zhang X. X. (2021). Benefits of runoff and sediment reduction under different ecological restoration modes on slopes of dump in arid mining area. J. Soil Water Conserv. 35, 15–21, 30. doi: 10.13870/j.cnki.stbcxb.2021.06.003

[B27] Yang X. W. Wang Y. Liu X. Yu H. X. Zhang Z. A. Tian Y. (2023). Development status and prospects of open-pit coal mines in China. China Coal 49, 126–133. doi: 10.19880/j.cnki.ccm.2023.06.018

[B28] Yang Y. Zhao W. W. Martinez-Murillo J. F. Pereira P. (2020). Loess Plateau: from degradation to restoration. Sci. Total Environ. 738, 140206. doi: 10.1016/j.scitotenv.2020.140206, PMID: 32660774

[B29] Yang Y. Zhao Y. N. Fan R. Y. Zhang Y. N. Liu L. Ding Y. (2024). A review of the effects of ecological restoration on soil organic carbon in grassland open-pit mines. Chinese Journal of Grassland 46 (09), 129–138. doi: 10.16742/j.zgcdxb.20240011

[B30] Yuan R. Y. (2024). Ecological stoichiometric characteristics of soil carbon, nitrogen and phosphorus under the canopies of *Haloxylon ammodendron* and *Kalidium caspicum*. Xinjiang Agricultural University, Urumqi. doi: 10.27431/d.cnki.gxnyu.2024.001209

[B31] Yuan Y. Q. Li Y. J. Wang X. Y. Meng W. C. Liu L. H. Yang F. (2025). Evolution of soil physicochemical properties and bacteria during vegetation restoration in southwest China coal gangue dump. J. Agro-Environment Sci., 1–14. Available online at: https://link.cnki.net/urlid/12.1347.S.20250819.1447.012.

[B32] Zhang Z. Y. Wu X. J. Liang Y. P. Zhang X. X. Zha T. G. (2023). Near-natural vegetation spatial allocation model for ecological restoration of abandoned mines in Wula Mountain. Arid Zone Res. 40, 1164–1171. doi: 10.13866/j.azr.2023.07.13

[B33] Zhao Z. H. Hao J. Li H. T. Xing L. T. Luo Z. J. Dong F. (2023). Ecological restoration of abandoned quarry wasteland under different restoration modes. J. Xinyang Normal Univ. (Natural Sci. Edition) 36, 262–268.

[B34] Zhou Y. Boutton T. W. Wu X. B. (2018). Soil phosphorus does not keep pace with soil carbon and nitrogen accumulation following woody encroachment. Global Change Biol. 24, 1992–2007. doi: 10.1111/gcb.14048, PMID: 29323781

[B35] Zhu G. Y. Zhang X. M. Yan X. H. . (2024). Evaluation of the construction process of modern energy system in China. Advanced Eng. Sci. 56, 206–217.

